# Structural, Magnetic, and Magnetocaloric Properties of Ce_2_(Fe, Co)_17_ Compounds: Tuning Magnetic Transitions and Enhancing Refrigeration Efficiency

**DOI:** 10.3390/ma18091958

**Published:** 2025-04-25

**Authors:** Hamdi Jaballah, Jihed Horcheni, Jacques Moscovici, Abderrahime Ayadim, Lotfi Bessais

**Affiliations:** Univ Paris Est Creteil, CNRS, ICMPE, UMR 7182, 2 Rue Henri Dunant, F-94320 Thiais, France; hamdi.jaballah@cnrs.fr (H.J.); jihed.horcheni@cnrs.fr (J.H.); jacques.moscovici@cnrs.fr (J.M.); ayadim@u-pec.fr (A.A.)

**Keywords:** intermetallic rare-earth transition metal compounds, magnetic materials, magnetocaloric effect

## Abstract

This study explores the structural, magnetic, and magnetocaloric properties of ${\mathrm{Ce}}_{2}$(Fe, Co${)}_{17}$ (x = 0, 0.5, 0.6, and 0.7) compounds synthesized via arc melting under high temperatures exceeding 2300 K. The as-cast ingots are subsequently sealed and subjected to a heat treatment at 1323 K to improve homogeneity and crystallinity. Detailed analyses using X-ray diffraction and magnetometry reveal that cobalt substitution significantly impacts the structural and magnetic behavior, enabling precise tuning of the magnetic transition temperature and magnetic order. The substitution induces an anisotropic increase in cell parameters and shifts the magnetocaloric effect (MCE) from low temperatures (200 K for x = 0) to near room temperature (285 K for x = 0.7), enhancing the operating temperature range. The magnetocaloric effect is studied across different magnetic transitions: a metamagnetic and ferro-antiferromagnetic transition followed by a paramagnetic state in one sample, and a direct ferro-paramagnetic transition in another. The compounds exhibit a second-order magnetic phase transition, ensuring a reversible MCE, with a relative cooling power (RCP) that is approximately 85% of that of pure Gd. Moreover, the use of cerium, the most cost-effective rare-earth element (5 $/kg), combined with its low atomic concentration (10%) in these intermetallics, enhances the sustainability and affordability of these materials. These findings underline the potential of iron-rich Ce-based compounds for next-generation refrigeration and energy-harvesting applications.

## 1. Introduction

The vapor-compression refrigeration technique, which has been employed for more than a century, remains the predominant cooling approach. Nevertheless, its efficiency is nearing its theoretical limit, and it accounts for a substantial share of global electricity usage and greenhouse gas emissions due to the reliance on high-impact refrigerants. This growing environmental concern has driven significant interest in eco-friendly alternatives, particularly solid-state materials capable of achieving cooling effects through adiabatic temperature variations or isothermal entropy modifications, offering a potential replacement for conventional vapor-compression refrigeration systems, which rely on hydrofluorocarbon (HFC) or hydrochlorofluorocarbon (HCFC) refrigerants.

In this framework, research efforts are increasingly directed toward solid-state materials that can exhibit adiabatic temperature variations or isothermal entropy fluctuations in response to external stimuli, aiming to establish a viable alternative to conventional vapor-compression cooling technologies.

Following the discovery of the magnetocaloric effect (MCE), extensive investigations have been conducted on a wide range of magnetocaloric materials. Among them, intermetallic compounds such as ${\mathrm{Gd}}_{5}$(Ge,Si${)}_{4}$ [1,2,3,4] have demonstrated promising performance due to their first-order transition and large entropy change. La(Fe,Co,Si${)}_{13}$-based hydrides [5,6,7,8,9,10] have attracted attention for their tunable transition temperatures and good refrigerant capacity, but they still suffer from hysteresis losses. Hexagonal ${\mathrm{Fe}}_{2}$P-type compounds [11,12,13,14] also stand out for their moderate MCE and reduced thermal hysteresis. Moreover, perovskite-type manganites $R_{1-x}A_{x}{\mathrm{MnO}}_{3}$ [15,16,17,18] have been thoroughly investigated due to their second-order magnetic transitions and chemical versatility.

Recent investigations have continued to explore the magnetocaloric characteristics of various materials, with a particular focus on optimizing performance in the vicinity of room temperature. For instance, Atanasov et al. [19] reported an enhanced MCE and tunable Curie temperatures in bulk and nano-sized ${\mathrm{Pr}}_{0.65-x}{\mathrm{Nd}}_{x}{\mathrm{Sr}}_{0.35}{\mathrm{MnO}}_{3}$ compounds, making them promising for room-temperature applications. In the context of amorphous thin films, Kumar et al. [20] demonstrated a broad operational window and “table-like” entropy change behavior in Gd-Fe-Co systems, suitable for Ericsson-cycle refrigeration. Monte Carlo simulations performed by Arejdal [21] on CrTe compounds confirmed a second-order magnetic transition with significant magnetocaloric properties near 326 K. Uporov and Sterkhov [22] investigated Gd-Sc alloys, revealing a wide MCE temperature span and high refrigerant capacity due to structural disorder. Furthermore, Luo et al. [23] reported a tunable and giant MCE in ${\mathrm{Mn}}_{x}{\mathrm{Fe}}_{2-x}P_{0.5}{\mathrm{Si}}_{0.5}$ microwires, which offers great potential for miniaturized magnetic refrigeration systems. Additionally, numerous review articles have been published summarizing recent advancements and outlining prospective developments in magnetocaloric materials and magnetic refrigeration systems [24,25].

Among elemental materials, gadolinium exhibits the most pronounced magnetocaloric effect near room temperature. However, its high susceptibility to oxidation, elevated cost, and limited global availability hinder its widespread application as a competitor to conventional cooling technologies. Compared to gadolinium and its derivatives, the iron-rich intermetallic compound $R_{2}{\mathrm{Fe}}_{17}$ is a cost-effective alternative. Extensive studies have been conducted on the magnetocaloric properties of this material class by chemical substitution. For instance, Datta et al. [26] demonstrated that partial substitution of Fe by Cr in ${\mathrm{Nd}}_{2}{\mathrm{Fe}}_{17-x}{\mathrm{Cr}}_{x}$ not only enhances thermal stability by reducing the magnetovolume effect but also maintains a relatively high Curie temperature ($T_{C}\approx 366$ K) and large relative cooling power (RCP). Similarly, Cengiz et al. investigated the influence of Cu [27] and Ti [27] substitutions in ${\mathrm{Pr}}_{2}{\mathrm{Fe}}_{17-x}M_{x}$ systems, reporting tunable $T_{C}$ values and second-order phase transitions with significant magnetocaloric performance, making these materials suitable for near-room-temperature applications.

Substitution of the rare-earth site has also been explored. Jaballah et al. [28] examined the (Pr,Sm${)}_{2}$${\mathrm{Fe}}_{17}$ system and observed that replacing Pr with Sm increases $T_{C}$ due to the de Gennes factor, improving magnetic entropy change near 300 K. Louhichi et al. [29] extended this approach to (Er,Nd${)}_{2}$${\mathrm{Fe}}_{17}$ compounds and achieved enhanced MCE through Er-to-Nd substitution, with Curie temperatures varying from 294 K to 335 K.

The effect of transition metal substitution on Gd-based compounds was studied by Saidi et al. [30], where Cr substitution in ${\mathrm{Gd}}_{2}{\mathrm{Fe}}_{17}$ notably altered the magnetic transition and enhanced the magnetocaloric effect, with RCP values up to 51.2 J/kg for x=1.5.

More recently, Horcheni et al. [31] investigated ${\mathrm{Pr}}_{2}{\mathrm{Fe}}_{16.9}{\mathrm{Ni}}_{0.1}$ and reported critical behavior consistent with a 3D Ising model, along with a moderate magnetic entropy change of 6.2 J/kg K around room temperature and an RCP close to 89% of that of Gd. Álvarez et al. [32] synthesized nanocrystalline $R_{2}{\mathrm{Fe}}_{17}$ (R = Pr, Nd) ribbons and observed double ferro-to-paramagnetic transitions due to grain boundary disorder, resulting in a broadened and “table-like” $\Delta S_{M}\left(T\right)$ curve and a substantial refrigerant capacity, which make them excellent candidates for room-temperature magnetic refrigeration.

Due to its relative abundance in the Earth’s crust, iron (Fe) is the most economical transition metal for magnetic refrigeration applications. Among rare-earth elements, cerium (Ce) is the most abundant and, consequently, the least expensive. According to the Shanghai Metals Market [33], the spot reference price of cerium metal was approximately 30,000 yuan/mt (around 4.5 $/kg based on the exchange rate in March 2021), which is significantly lower than that of other rare-earth elements, such as praseodymium (650,000 yuan/mt, about 98 $/kg), neodymium (100 $/kg), or dysprosium (300 $/kg). These two factors—the low costs of Fe and Ce—make Fe-rich Ce-Fe compounds highly attractive candidates for cost-effective magnetic refrigeration materials.

In this study, we focus on ${\mathrm{Ce}}_{2}{\mathrm{Fe}}_{17}$, though the expected favorable conditions are not entirely satisfied. The most Fe-rich Ce-Fe compound, ${\mathrm{Th}}_{2}{\mathrm{Zn}}_{17}$-type rhombohedral ${\mathrm{Ce}}_{2}{\mathrm{Fe}}_{17}$, exhibits unconventional magnetic behavior. To induce the desired ferromagnetic state instead of its inherent heli-magnetic nature, we propose substituting iron with cobalt.

In this work, we investigate the structural, magnetic, and magnetocaloric characteristics near the magnetic phase transition.

## 2. Experimental Methods

To prepare the intermetallic compound ${\mathrm{Ce}}_{2}$(Fe, Co${)}_{17}$, meticulous steps were followed using high-purity elements. Firstly, the elements were melted in an arc furnace, resulting in the formation of an ingot. The local temperature during arc melting typically exceeds 2000 °C to ensure the complete melting and homogenization of the constituent elements. To safeguard its integrity, the ingot was enveloped with tantalum foil and hermetically sealed within a silica tube under a secondary vacuum. The sealed samples underwent a heat treatment process at a temperature of 1323 K for a duration of seven days, followed by a rapid cooling process through water quenching. This methodology ensured the attainment of high purity and uniformity in all samples, which are essential for an accurate investigation of their properties. By utilizing tantalum foil and silica tube sealing under a secondary vacuum, the sample was shielded from any potential contamination during the heat treatment process. The heat treatment and water-quenching steps played a crucial role in stabilizing the crystal structure and enhancing the quality of the sample.

Furthermore, X-ray diffraction (XRD) was employed as a characterization technique to analyze the crystal structure of the ${\mathrm{Ce}}_{2}{\mathrm{Fe}}_{17-x}{\mathrm{Co}}_{x}$ samples. The XRD patterns were carried out on a Bruker D8 diffractometer with Cu-Kα radiation λ=1.5405. XRD data of the sample were collected between 2θ=15 and 2θ=100 at room temperature with 0.02 step width and counting times of 6s per point. The refinement of the pattern was performed using the Rietveld method [34] as implemented in the FullProf computer software [35]. This technique furnished detailed information about the lattice parameters, crystal structure, and phase purity of the samples.

In order to study the magnetic properties of ${\mathrm{Ce}}_{2}{\mathrm{Fe}}_{17-x}{\mathrm{Co}}_{x}$, magnetization measurements were carried out using a Physical Property Measurement System (PPMS). This system enabled precise measurements of the magnetic behavior of ${\mathrm{Ce}}_{2}{\mathrm{Fe}}_{17-x}{\mathrm{Co}}_{x}$ under diverse conditions, including varied temperatures and magnetic fields. The experiments were conducted under external magnetic fields $H_{a}$ ranging from 0 to 5 T. To determine the Curie temperature ($T_{C}$), temperature-dependent magnetization curves M(T) were recorded in the range 10–340 K with a temperature step of 5 K under a constant applied field of $\mu_{0}H_{a}=0.05\;T$. Additionally, isothermal magnetization curves M(H) were obtained to study the field dependence of the magnetic response. Accurate and high-resolution data were essential for extracting meaningful insights into the critical behavior. Around the Curie temperature, M(H) measurements were taken with temperature intervals of 4 K, while a larger step of 6 K was used for the composition with x=0. The applied field was incremented in constant steps of $\mu_{0}H=0.1\;T$ between 0 and 1 T, and $\mu_{0}H=0.5\;T$ from 1 to 5 T. The magnetocaloric effect (MCE) was subsequently derived as a function of temperature and magnetic field through numerical integration of the isothermal M(H) curves.

## 3. Results and Discussions

### 3.1. Structural Properties

#### 3.1.1. Crystal Structure

**Figure 1:**
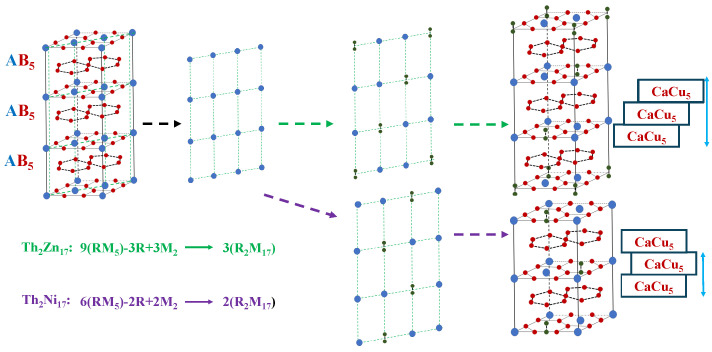
Crystal structures: ${\mathrm{Th}}_{2}{\mathrm{Ni}}_{17}$-type structure with $P6_{3}/mmm$ space group and ${\mathrm{Th}}_{2}{\mathrm{Zn}}_{17}$-type structure with $R\bar{3}m$ space group.

$R_{2}{\mathrm{Fe}}_{17}$ compounds exhibit distinct crystal structures, which can be discerned through analysis of their powder diffraction patterns. When the rare-earth element R is Pr, Nd, or Sm, these compounds are commonly observed to crystallize in the rhombohedral structure known as ${\mathrm{Th}}_{2}{\mathrm{Zn}}_{17}$. On the other hand, for R = Gd, Tb, Dy, Ho, Er, Tm, or Lu, the compounds adopt a hexagonal structure referred to as ${\mathrm{Th}}_{2}{\mathrm{Ni}}_{17}$ (see Figure 1).

In the rhombohedral ${\mathrm{Th}}_{2}{\mathrm{Zn}}_{17}$ structure, each unit cell comprises nine entities. This arrangement involves the replacement of three rare-earth atoms (R) by an M-M dumbbell at a specific site referred to as the dumbbell site. This substitution process can be represented by the following equation: $9\left(RM_{5}\right)-3R+3M_{2}\to 3R_{2}M_{17}$. The ${\mathrm{Th}}_{2}{\mathrm{Zn}}_{17}$ crystal structure belongs to the space group $R\bar{3}m$ and offers different sites for the rare-earth atoms (R) and M atoms, denoted by their Wyckoff notation (6c, 9d, 18f, 18h). The R atoms occupy a single 6c site, while the M atoms are distributed across the other available sites.

In contrast, the hexagonal Th_2_Ni_17_ structure consists of six entities per unit cell. In this arrangement, two of the rare-earth atoms (R) are replaced by an M-M at the dumbbell site. The substitution is represented by the following equation: $6\left(RM_{5}\right)-2R+2M_{2}\to 2R_{2}M_{17}$. The ${\mathrm{Th}}_{2}{\mathrm{Ni}}_{17}$ crystal structure adopts the space group $P6_{3}/mmm$, and, similar to the ${\mathrm{Th}}_{2}{\mathrm{Zn}}_{17}$ structure, it provides specific sites for the rare-earth atoms (R) and M atoms. The M atoms occupy four consistent sites (4f, 6g, 12j, and 12k), while the R atoms can be found at two available sites (2b and 2c).

It is noteworthy that, within the ${\mathrm{Th}}_{2}{\mathrm{Zn}}_{17}$ structure, the dumbbell configuration of the M atoms exhibits symmetry with respect to the basal plane containing the rare-earth atoms (R). This arrangement of the M atoms contributes to the overall stability and structural integrity of the crystal.

**Figure 2:**
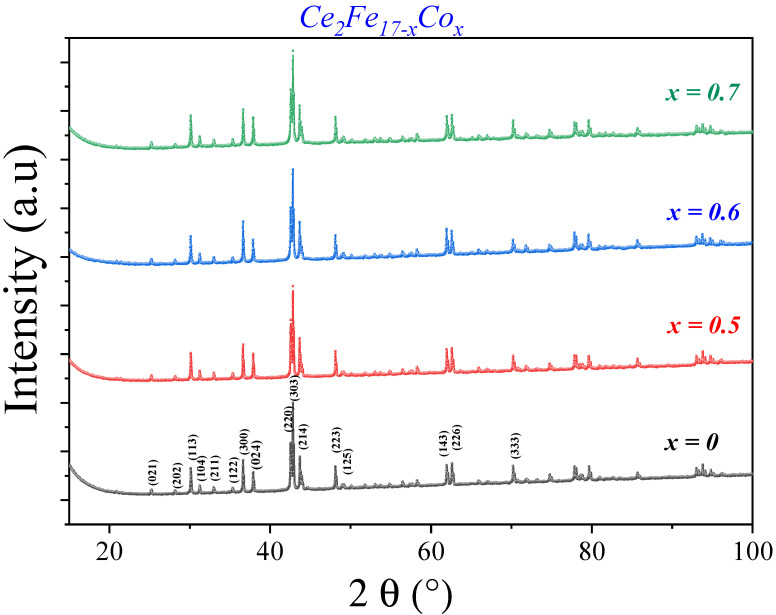
X-ray diffraction of ${\mathrm{Ce}}_{2}{\mathrm{Fe}}_{17-x}{\mathrm{Co}}_{x}$ compounds as a function of cobalt substitution.

Figure 2 shows the X-ray diffraction of ${\mathrm{Ce}}_{2}{\mathrm{Fe}}_{17-x}{\mathrm{Co}}_{x}$ compounds as a function of cobalt substitution annealed at 1323 K for 7 days. For cobalt contents ranging from 0 to 0.7, all the angular positions of the main peaks show a single ${\mathrm{Th}}_{2}{\mathrm{Zn}}_{17}$ crystallographic structure of space group $R\bar{3}m$. The substitution of iron with cobalt does not lead to a change in the structure.

#### 3.1.2. Rietveld Refinement

**Figure 3:**
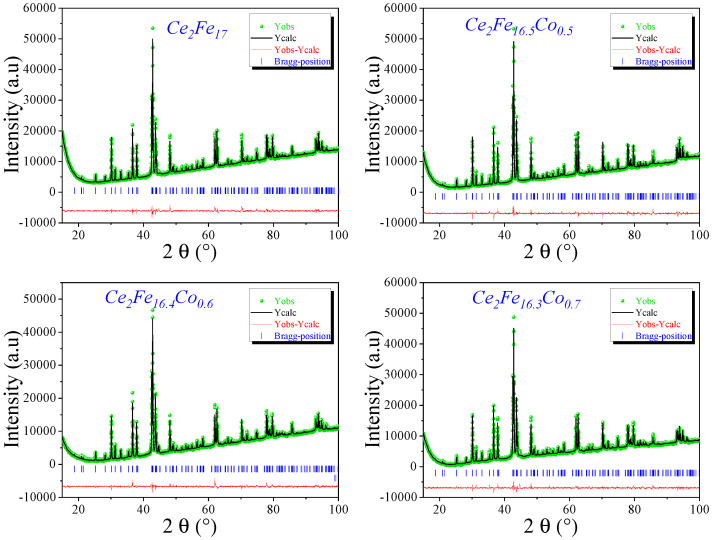
The Rietveld refinement pattern of XRD data for ${\mathrm{Ce}}_{2}{\mathrm{Fe}}_{17-x}{\mathrm{Co}}_{x}$ compounds. The green symbols represent the measured intensities, while the black line depicts the computed intensities. The blue vertical bars denote the (hkl) Bragg peak positions. The red curve illustrates the discrepancy between experimental and calculated intensities.

Following the X-ray diffraction, a Rietveld refinement of the diffraction patterns is essential to determine the lattice parameters and assess the effect of Co substitution on the structure. The refinement results, illustrated in Figure 3, confirm that the crystal structure remains unchanged even after Co substitution. In these diffraction profiles, the experimental data (green symbols) align well with the calculated intensities (black line), while the blue vertical bars indicate the (hkl) Bragg peak positions. The red curve, representing the difference between measured and calculated intensities, demonstrates a satisfactory refinement quality.

Despite the preservation of the overall structure, a slight variation in the lattice parameters is observed with increasing Co concentration. Table 1 presents the refined structural parameters, including the lattice constants (*a* and *c*), unit cell volume (*V*), and refinement quality indicators ($R_{B}$ and $\chi^{2}$). The lattice parameter *a* remains relatively stable across all compositions, showing only minor fluctuations, whereas the *c* parameter exhibits a subtle but systematic increase with Co content. Consequently, the c/a ratio slightly increases from 1.4619 for ${\mathrm{Ce}}_{2}{\mathrm{Fe}}_{17}$ to 1.4640 for ${\mathrm{Ce}}_{2}{\mathrm{Fe}}_{16.3}{\mathrm{Co}}_{0.7}$, reflecting a slight elongation along the *c*-axis.

Interestingly, the unit cell volume *V* remains nearly constant, varying only from $775.8\;{Å}^{3}$ for x=0 to $778.0\;{Å}^{3}$ for x=0.7, suggesting that Co substitution does not induce significant volumetric expansion. This behavior indicates that the replacement of Fe by Co is accommodated within the structure without causing major distortions, preserving the integrity of the 2:17 *R*-type phase.

Although these structural variations appear minor, they can have a non-negligible influence on the magnetic properties. The slight increase in the c/a ratio can enhance the uniaxial anisotropy, which plays a crucial role in the magnetocrystalline anisotropy of these compounds. Additionally, the nearly constant volume suggests that the dominant factor influencing the magnetic behavior is not a structural expansion but rather an electronic effect induced by Co substitution.

From an electronic perspective, Co has one additional electron compared to Fe, which significantly affects the electronic band structure and magnetic interactions. The presence of Co alters the hybridization between 3d transition metal states and Ce 4f orbitals, potentially modifying the strength of exchange interactions. This electronic effect can contribute to the observed enhancement of the Curie temperature ($T_{C}$) with increasing Co content, as previously discussed. Furthermore, the substitution of Fe by Co can influence the balance between ferromagnetic and antiferromagnetic interactions, leading to a more stable magnetic phase at higher temperatures.

Overall, these findings highlight the advantages of Co substitution in fine-tuning the structural and magnetic properties of ${\mathrm{Ce}}_{2}{\mathrm{Fe}}_{17}$-based compounds. While the structural modifications remain minimal, the electronic contribution of Co plays a significant role in enhancing the magnetic performance.

### 3.2. Magnetic Properties

#### 3.2.1. Thermomagnetic Properties

The thermomagnetic properties of the investigated samples were analyzed through temperature-dependent magnetization measurements (M−T) under an applied magnetic field of 0.05T. These measurements were conducted under both field-cooled conditions to capture the thermal evolution of magnetization.

**Figure 4:**
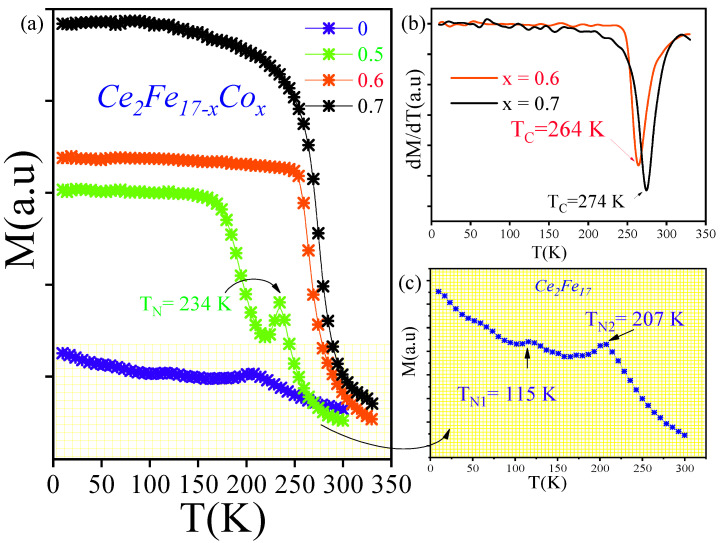
(**a**) *M* vs. *T* for all compositions of ${\mathrm{Ce}}_{2}{\mathrm{Fe}}_{17-x}{\mathrm{Co}}_{x}$. (**b**) First derivative of magnetization as a function of *T* for the compositions with x=0.6 and x=0.7. (**c**) *M* vs. *T* for ${\mathrm{Ce}}_{2}{\mathrm{Fe}}_{17}$ with enhanced clarity.

The *M*–*T* curves presented in Figure 4a indicate that the parent ${\mathrm{Ce}}_{2}{\mathrm{Fe}}_{17}$ compound undergoes two distinct magnetic transitions within the examined temperature range of 10–300 K. Below 115 K, the material exhibits an antiferromagnetic state, transitioning to a helical magnetic state in the range of 115–207 K before reaching a paramagnetic state above 215 K. These observations align with the known magnetic behavior of the binary ${\mathrm{Ce}}_{2}{\mathrm{Fe}}_{17}$ phase, as reported in previous studies like that of Givord et al. [36], who suggested that the magnetic structure of ${\mathrm{Ce}}_{2}{\mathrm{Fe}}_{17}$ evolves through several distinct configurations. At low temperatures, the system adopts a fan-like magnetic arrangement, which transitions to a helical configuration near 110 K and subsequently enters a paramagnetic regime above 210 K. However, this interpretation has been challenged by Plumier et al [37] as well as by Janssen et al. [38,39], who found no evidence supporting the presence of a fan structure in the ground state. Instead, they propose that the magnetic ground state is better described by a modified helical arrangement that lacks any ferromagnetic component.

After substituting with cobalt at x=0.5, a single antiferromagnetic-to-paramagnetic (AFM-PM) transition is observed at $T_{N}=234$ K. This indicates the disappearance of the first AFM-PM transition previously detected at lower temperatures in the parent compound.

In contrast, Co-substituted variants with x=0.6 and 0.7 exhibit a single pronounced transition from a ferromagnetic to a paramagnetic state (FM-PM), suggesting that the incorporation of Co significantly modifies the magnetic transition mechanisms of the 2:17 *R* matrix phase. For these two compositions, the M−T curves were analyzed, and the Curie temperature $T_{C}$ was determined by identifying the minimum in the first derivative of the M−T curves.

Figure 4b illustrates that $T_{C}$ increases from 265 K to 275 K as the Co concentration rises from x=0.6 to x=7. Consequently, Co substitution appears to enhance the magnetic interactions, leading to an overall increase in the Curie temperature ($T_{C}$) from $T_{N}=210$ K for x=0 to $T_{C}=275$ K for x=0.7.

These findings indicate that Co doping effectively shifts $T_{C}$ to higher temperatures and sharpens the nature of the phase transition, thereby improving the thermal stability of the magnetic phase.

#### 3.2.2. Isothermal Magnetization Curves and Phase Transition

To gain deeper insight into the field dependence of the magnetic phase transition in our compounds, isothermal magnetization measurements M(T−H) were performed.

**Figure 5:**
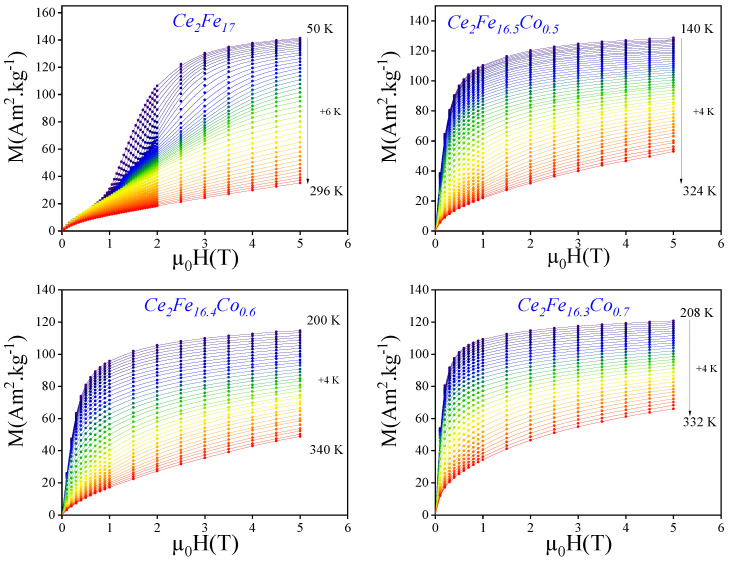
The variation of magnetization *M* as a function of the applied magnetic field $\mu_{0}H$ for the ${\mathrm{Ce}}_{2}{\mathrm{Fe}}_{17-x}{\mathrm{Co}}_{x}$ series (x=0,0.5,0.6, and 0.7).

Figure 5 presents the variation of magnetization *M* as a function of the applied magnetic field $\mu_{0}H$ for the ${\mathrm{Ce}}_{2}{\mathrm{Fe}}_{17-x}{\mathrm{Co}}_{x}$ series with x=0,0.5,0.6, and 0.7. The magnetization curves reveal a systematic evolution with increasing Co content. For the parent compound (x=0), the magnetization increases progressively with the applied field only when H>2 T, exhibiting a characteristic behavior of a ferromagnetic system. At low applied fields, the magnetization *M* increases with temperature under a constant magnetic field. Conversely, at higher fields, the trend reverses, highlighting the antiferromagnetic nature of the compound.

As Co is introduced into the structure, a noticeable enhancement in magnetization is observed, particularly for x=0.6 and x=0.7. This increase suggests a strengthening of the ferromagnetic interactions due to the substitution of Fe by Co, which is known to enhance magnetic exchange interactions. Furthermore, the saturation magnetization appears to rise with increasing Co concentration, indicating a modification of the intrinsic magnetic properties of the ${\mathrm{Ce}}_{2}{\mathrm{Fe}}_{17}$ matrix. These findings align with the previously observed trend in the Curie temperature, where Co doping was found to shift $T_{C}$ to higher temperatures, further supporting the enhancement of magnetic interactions in the system.

**Figure 6:**
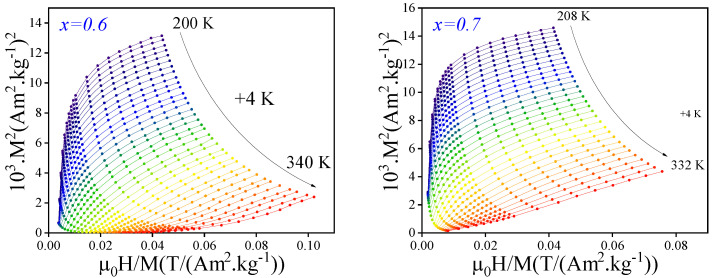
The Arrott plots of the ${\mathrm{Ce}}_{2}{\mathrm{Fe}}_{17-x}{\mathrm{Co}}_{x}$ alloys with x=0.6 and 0.7.

The magnetic (M−H−T) data for the compositions x=0.5, 0.6, and 0.7 were analyzed using Arrott plots, which involve plotting $M^{2}$ as a function of $\mu_{0}H/M$ to determine the nature of the magnetic phase transition, as illustrated in Figure 6. The positive slope of the Arrott plots confirms the presence of a second-order phase transition in these alloys. According to Banerjee’s criterion [40], a positive slope in the Arrott plots indicates a second-order ferromagnetic-to-paramagnetic (FM-PM) phase transition. This characteristic is particularly advantageous for magnetic materials, as second-order transitions ensure a continuous evolution of the magnetic entropy, whereas first-order transitions induce abrupt and discontinuous changes. Such a behavior is essential for applications requiring precise control of magnetic properties, including magnetocaloric and spintronic devices.

#### 3.2.3. Magnetocaloric Effect

The magnetic softness of ${\mathrm{Ce}}_{2}{\mathrm{Fe}}_{17-x}{\mathrm{Co}}_{x}$ compounds prompted us to assess its magnetocaloric characteristics. The calculation of magnetic entropy change based on the Maxwell relation is an important quantity in the field of magnetism as it provides information about the thermal energy and magnetic properties of a material. The magnetic entropy change ($\Delta S_{M}$) values were derived from the isothermal magnetization curves M(H) for the Co-substituted compounds with x=0.5, 0.6, and 0.7, in the vicinity of the magnetic transition temperature. We used calculation software based on an integral instead of a summation to obtain the value of $\Delta S_{M}$ at different fields and temperatures according to the following equation [41,42]:

$$\Delta S_{M}\left(\frac{T_{1}+T_{2}}{2}\right)=\frac{1}{T_{1}-T_{2}}\times \int_{0}^{H_{\max}}M\left(T_{2},\mu_{0}H\right)\mu_{0}dH-\int_{0}^{H_{\max}}M\left(T_{1},\mu_{0}H\right)\mu_{0}dH$$

where $\Delta S_{M}$ is the magnitude of the magnetic entropy change, and $\mu_{0}$ is the permeability of free space. This formula expresses the relationship between the magnetic entropy change and the magnetic properties of a material. The magnetic moment of a material is a measure of its response to changes in a magnetic field and is dependent on temperature. The magnetic entropy change reflects the change in the thermal energy of the material as a result of changes in a magnetic moment and magnetic field. It provides a quantitative measure of the degree of magnetic ordering in the material and is an important factor in the study of magnetic refrigeration.

**Figure 7:**
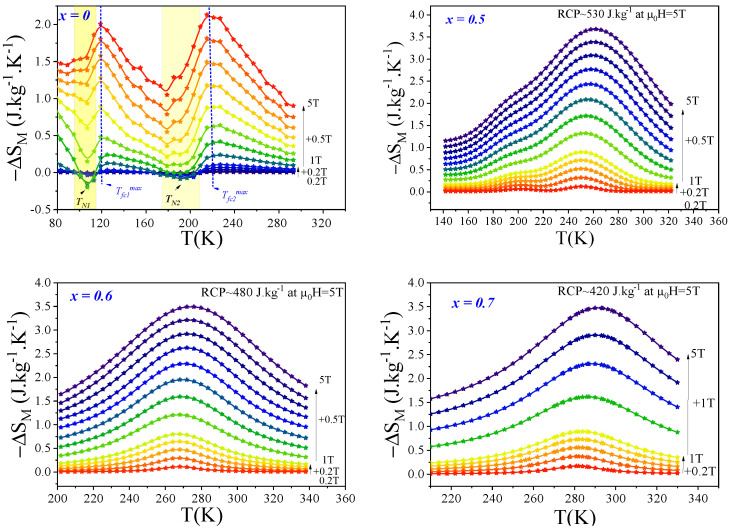
The isothermal entropy change $\Delta S_{M}$ was determined using the integrated Maxwell relation for a magnetic field variation of $\mu_{0}\Delta H=0-5$ T for ${\mathrm{Ce}}_{2}{\mathrm{Fe}}_{17-x}{\mathrm{Co}}_{x}$ compounds with (x=0,0.5,0.6, and 0.7).

Figure 7 shows the temperature dependence of the magnetic entropy change ($-\Delta S_{M}$) for ${\mathrm{Ce}}_{2}{\mathrm{Fe}}_{17-x}{\mathrm{Co}}_{x}$ compounds under different applied magnetic fields. As expected, the magnitude of $-\Delta S_{M}$ increases with the applied field. Notably, some compositions exhibit broad and symmetric peaks, indicating a second-order magnetic transition. A distinct behavior is observed for the parent compound (x = 0), which displays two peaks, suggesting the presence of two magnetic transitions at different temperatures.

Another essential factor to consider while selecting magnetocaloric materials is the relative cooling power (RCP), defined by Gschneidner and Pecharsky [43] as the amount of heat transferred between the hot and cold sides in an ideal refrigeration cycle. It is a key parameter used to evaluate the magnetocaloric efficiency. The RCP was computed using the following relation [44]:

(1) $$RCP=|-\Delta S_{M}^{\max}|\times \delta T_{\mathrm{FWHM}}$$

where $-\Delta S_{M}^{\max}$ is the maximum magnetic entropy change, and $\delta T_{\mathrm{FWHM}}$ is the full width at half maximum of the $-\Delta S_{M}\left(T\right)$ peak.

Table 2 highlights the magnetocaloric performances of ${\mathrm{Ce}}_{2}{\mathrm{Fe}}_{17-x}{\mathrm{Co}}_{x}$ compounds under various applied magnetic fields and compares them with other well-known magnetocaloric materials. Both the $-\Delta S_{M}^{\max}$ and RCP increase significantly with increasing magnetic field. For instance, ${\mathrm{Ce}}_{2}{\mathrm{Fe}}_{16.5}{\mathrm{Co}}_{0.5}$ exhibits a $-\Delta S_{M}^{\max}$ of 3.7 J/(kg·K) and an RCP of 533 J/kg at 5 T.

The parent compound ${\mathrm{Ce}}_{2}{\mathrm{Fe}}_{17}$ (x = 0) stands out due to its two distinct operating temperatures at approximately 120 K and 200 K under a 2 T field. Although its $-\Delta S_{M}^{\max}$ values are modest (0.25 and 0.4 J/(kg·K)), the corresponding $\delta T_{\mathrm{FWHM}}$ values of 40 K and 80 K, respectively, lead to an extended operational range. Under 5 T, the entropy change increases to 2 and 2.1 J/(kg·K), with an RCP exceeding 400 J/kg, making it a promising candidate for multi-stage magnetic refrigeration systems.

Among the Co-substituted compounds, ${\mathrm{Ce}}_{2}{\mathrm{Fe}}_{16.4}{\mathrm{Co}}_{0.6}$ and ${\mathrm{Ce}}_{2}{\mathrm{Fe}}_{16.3}{\mathrm{Co}}_{0.7}$ provide a favorable compromise between $-\Delta S_{M}^{\max}$ and operating temperature, with RCP values of 480 J/kg at 280 K and 420 J/kg at 290 K, respectively. These values are comparable or superior to those of other compounds, such as ${\mathrm{Nd}}_{2}{\mathrm{Fe}}_{17}$ (RCP = 105 J/kg at 339 K), ${\mathrm{Sm}}_{0.36}{\mathrm{Pr}}_{1.64}{\mathrm{Fe}}_{17}$ (RCP = 247 J/kg at 300 K), and ${\mathrm{Pr}}_{2}{\mathrm{Fe}}_{16.9}{\mathrm{Ni}}_{0.1}$ (RCP = 508 J/kg at 300 K).

While pure Gd remains one of the most studied materials, with a high $-\Delta S_{M}^{\max}$ of 4.8 J/(kg·K) and an RCP of 213 J/kg at 293 K, the ${\mathrm{Ce}}_{2}{\mathrm{Fe}}_{17-x}{\mathrm{Co}}_{x}$ series offers more tunable properties over broader temperature ranges, making them highly relevant for specific applications.

These results confirm the potential of these alloys as efficient magnetocaloric materials, especially for room-temperature or multi-stage cooling technologies.

From a technological standpoint, the laboratory-scale synthesis of ${\mathrm{Ce}}_{2}{\mathrm{Fe}}_{17-x}{\mathrm{Co}}_{x}$ compounds has already demonstrated good structural stability and reproducibility with optimized synthesis conditions. However, for large-scale industrial production, certain challenges may arise, particularly related to the control of the melting temperature in industrial furnaces. Indeed, the relatively high volatility of cerium at elevated temperatures may lead to cerium losses and stoichiometric deviations during the melting process, which requires careful control and optimization of the processing parameters. Apart from this aspect, the investigated materials exhibit stable magnetic properties and phase purity, which are essential for practical applications.

Moreover, the development of magnetocaloric refrigeration devices based on these compounds could represent an eco-friendly alternative to conventional vapor-compression systems. Although the initial cost might be higher due to the need for permanent magnets to generate the magnetic field and the material quantity required, such systems offer the advantage of reduced energy consumption and the elimination of greenhouse gas emissions, aligning with sustainable and environmentally friendly technologies for future refrigeration applications.

## 4. Conclusions

In this study, we have thoroughly investigated the structural, magnetic, and magnetocaloric properties of ${\mathrm{Ce}}_{2}{\mathrm{Fe}}_{17-x}{\mathrm{Co}}_{x}$ compounds (x=0, 0.5, 0.6, 0.7). X-ray diffraction and Rietveld refinement analyses confirmed that all synthesized samples crystallized in the rhombohedral ${\mathrm{Th}}_{2}{\mathrm{Zn}}_{17}$-type structure, and cobalt substitution does not induce any structural phase transition. However, slight variations in the lattice parameters, especially the c/a ratio, reflect subtle structural adjustments upon Co doping. Magnetization measurements from 10 K to 340 K revealed that the parent compound ${\mathrm{Ce}}_{2}{\mathrm{Fe}}_{17}$ exhibits complex magnetic behavior with two successive transitions: an antiferromagnetic (AFM) ground state followed by a helical configuration before becoming paramagnetic at high temperatures. Remarkably, cobalt substitution progressively suppresses the AFM state and stabilizes a ferromagnetic (FM) ground state. For x=0.6 and x=0.7, the compounds exhibited a single sharp FM-PM transition, confirming that Co doping effectively transforms the magnetic nature from AFM to FM. This magnetic tunability highlights the strong potential of chemical substitution strategies to control the magnetic properties of $R_{2}$${\mathrm{Fe}}_{17}$-type intermetallics. Furthermore, the magnetocaloric effect of the investigated materials was significantly enhanced by cobalt doping. The presence of a ferromagnetic ordering, combined with a sharp magnetic transition, favors a more efficient magnetocaloric response.

Overall, the ${\mathrm{Ce}}_{2}{\mathrm{Fe}}_{17-x}{\mathrm{Co}}_{x}$ system, especially for intermediate cobalt contents, presents an excellent compromise between structural stability, magnetic tunability, and enhanced magnetocaloric properties. These features reinforce the potential of these low-cost, iron-rich materials as promising and environmentally friendly alternatives to gadolinium-based refrigerants, paving the way towards the development of efficient and sustainable solid-state cooling technologies.

Beyond the scope of this study, further investigations could focus on optimizing the synthesis process to enhance the material’s homogeneity and mechanical properties, which are crucial for large-scale applications. Moreover, the strategy of magnetic tuning by transition metal substitution could be extended to other rare-earth-based intermetallic compounds with similar structures, opening new pathways for the design of advanced materials for energy-efficient refrigeration technologies or other magnetically driven devices.

## Figures and Tables

**Table 1:** Lattice parameters (*a* and *c*), unit cell volume (*V*), refinement quality indicators ($R_{B}$, $\chi^{2}$), and atomic positions obtained from Rietveld refinement for ${\mathrm{Ce}}_{2}{\mathrm{Fe}}_{17}$ and its Co-substituted derivatives.

	${\mathrm{Ce}}_{2}{\mathrm{Fe}}_{17}$	${\mathrm{Ce}}_{2}{\mathrm{Fe}}_{16.5}{\mathrm{Co}}_{0.5}$	${\mathrm{Ce}}_{2}{\mathrm{Fe}}_{16.4}{\mathrm{Co}}_{0.6}$	${\mathrm{Ce}}_{2}{\mathrm{Fe}}_{16.3}{\mathrm{Co}}_{0.7}$
*a* (Å)	8.4938(1)	8.4931(1)	8.4956(1)	8.4965(1)
*c* (Å)	12.4178(1)	12.4255(1)	12.4321(1)	12.4390(1)
c/a ratio	1.4619	1.4626	1.4633	1.4640
*V* (${Å}^{3}$)	775.8	776.2	777.1	778.0
$\chi^{2}$	2.19	2.37	1.77	1.69
$R_{B}$	5.72	6.88	5.46	5.39
Atomic Coordinates				
*x* (Fe, 18f site)	0.2907(3)	0.2908(2)	0.2917(2)	0.2920(2)
*x* (Fe/Co, 18h site)	0.497(1)	0.477(2)	0.5028(2)	0.5050(2)
*z* (Ce, 6c site)	0.344(1)	0.344(1)	0.3439(2)	0.3439(2)
*x* (Fe, 6c site)	0.0956(3)	0.0958(1)	0.092(2)	0.0915(2)
*z* (Fe/Co, 18h site)	0.1549(2)	0.155(1)	0.166(2)	0.172(2)

**Table 2:** Values of applied magnetic field $\mu_{0}\Delta H$, operating temperature $T_{fc}^{max}$, maximum of entropy variation $|-\Delta S_{M}^{\max}|$, and relative cooling power (RCP) of ${\mathrm{Ce}}_{2}{\mathrm{Fe}}_{17-x}{\mathrm{Co}}_{x}$ system compared with other magnetic materials.

Sample	$\mu_{0}\Delta H$	$T_{\mathrm{fc}}^{\max}$	$-\Delta S_{M}^{\max}$	$\delta T^{\mathrm{FWHM}}$	RCP	Ref
	(T)	(K)	J/(kg·K)	(K)	(J/kg)	
${\mathrm{Ce}}_{2}{\mathrm{Fe}}_{17}$	2	120;200	0.25;0.4	40;80	10;32	This work
	5		2;2.1	>200	>400	
${\mathrm{Ce}}_{2}{\mathrm{Fe}}_{16.5}{\mathrm{Co}}_{0.5}$	2	255	1.7	110	187	-
	5	262	3.7	144	533	-
${\mathrm{Ce}}_{2}{\mathrm{Fe}}_{16.4}{\mathrm{Co}}_{0.6}$	2	267	1.6	90	144	-
	5	280	3.5	137	480	-
${\mathrm{Ce}}_{2}{\mathrm{Fe}}_{16.3}{\mathrm{Co}}_{0.7}$	2	285	1.6	88	140	-
	5	290	3.5	120	420	-
${\mathrm{Nd}}_{2}{\mathrm{Fe}}_{17}$	1.5	339	1.7	50	105	[45]
${\mathrm{Sm}}_{0.36}{\mathrm{Pr}}_{1.64}{\mathrm{Fe}}_{17}$	3	300	3.24	78	247	[28]
${\mathrm{Ce}}_{1.6}{\mathrm{Nd}}_{0.4}{\mathrm{Fe}}_{17}$	5	273	4.1	90.5	371	[46]
${\mathrm{Ce}}_{1.6}{\mathrm{Nd}}_{0.4}{\mathrm{Fe}}_{16.67}{\mathrm{Si}}_{0.33}$	5	297	3.7	94.5	350	[46]
${\mathrm{Pr}}_{2}{\mathrm{Fe}}_{16.9}{\mathrm{Ni}}_{0.1}$	5	300	6.2	82	508	[31]
$Y_{2}{\mathrm{Fe}}_{17}$	5	303	4.6	103	478	[47]
${\mathrm{Er}}_{2}{\mathrm{Fe}}_{17}$	5	290	3.5	67	234.5	[29]
${\mathrm{Gd}}_{2}{\mathrm{Fe}}_{17}$	1.5	475	1.2	15	18	[48]
Gd	2	293	4.8	44	213	[1]

## Data Availability

The original contributions presented in this study are included in the article. Further inquiries can be directed to the corresponding author.
